# Editorial: Advancements and challenges in veterinary oncology

**DOI:** 10.3389/fvets.2026.1787676

**Published:** 2026-02-03

**Authors:** Felisbina Queiroga, Rodrigo dos Santos Horta, Antonio Giuliano, Maria Lúcia Zaidan Dagli

**Affiliations:** 1 Centro de Ciência Animal e Veterinária (CECAV), University of Trás-os-Montes and Alto Douro, Vila Real, Portugal; 2 Department of Veterinary Medicine and Surgery, Veterinary School, Universidade Federal de Minas Gerais (UFMG), Belo Horizonte, Minas Gerais, Brazil; 3 Harvest Veterinary Oncology Center (HVOC), Hong Kong, Hong Kong SAR, China; 4 Department of Pathology, School of Veterinary Medicine and Animal Science, University of São Paulo (USP), São Paulo, Brazil

**Keywords:** cancer, guideline, liquid biopsy, precision medicine, tumor biology

Veterinary oncology has undergone remarkable transformation over the past decade, driven by rapid advances in molecular biology, imaging, therapeutics, and translational research (1). These developments have substantially expanded diagnostic precision and therapeutic possibilities for companion animals with cancer. Despite this progress, significant challenges remain, including the identification of reliable prevention and early detection strategies, optimization of multimodal treatments and integration of molecular and genetic data into clinical decision-making (2, 3). This Research Topic, *Advancements and Challenges in Veterinary Oncology*, includes 12 contributions that reflect the breadth of contemporary veterinary oncology, spanning clinical management, translational research, molecular oncology, and evidence-based guidelines.

A major thematic pillar across the published articles relates to diagnostics and tumor characterization, including the need to improve how tumors are detected, assessed, and classified. Ko et al. combined circulating cell-free DNA profiling with machine learning for early detection of canine hemangiosarcoma, highlighting both the potential and current limitations of liquid biopsy in aggressive, heterogeneous tumors. Within the diagnostic and biomarker axis, Rösch et al. evaluated serum survivin, Ki-67, thymidine kinase 1, and the survivin–lymphocyte ratio in dogs with chronic nasal disease. While not diagnostic individually, survivin-related markers were increased in malignant cases, supporting their potential role in early suspicion and monitoring, while underscoring the overlap between neoplastic and inflammatory conditions. Vincenti et al. addressed a highly practical and widely relevant issue by evaluating combined cross-sectional and tangential surgical margin assessment in dogs and cats with different tumor types. Their findings highlight the risk of underestimating margin involvement when relying on a single trimming approach and support more standardized and informative pathology reporting, with direct implications for surgical planning and post-operative management. Díaz-Santana et al. reported a case of metastatic renal giant-cell sarcoma in an African pygmy hedgehog, providing detailed pathological and immunohistochemical characterization of an uncommon studied species. Although case reports are not designed to change practice on their own, they strengthen the collective knowledge base, support comparative pathology, and remind us that oncological challenges extend beyond the classical canine and feline settings. The Research Topic also includes work directly aligned with the development of immunology-enabling tools, which are essential for both research and clinical translation. Li et al. reported the preparation and functional validation of a rabbit anti-canine CD3 monoclonal antibody, addressing a practical methodological barrier in canine immunophenotyping and immune profiling. The availability of validated reagents of this nature is fundamental for consistent immunohistochemical analyses and for advancing the study of tumor immune microenvironments in veterinary species.

Beyond primary diagnostics, the Research Topic also addresses tumor characterization and disease-specific overviews that help clinicians interpret heterogeneous presentations and rationally select interventions. Marcinowska et al. provided a descriptive review of canine lung carcinoma, summarizing etiology, clinical presentation, staging, and current treatment options, while framing persistent controversies around adjuvant therapies. Importantly, the review reinforces that surgery remains the cornerstone of treatment when feasible, while pointing to emerging targeted and immunotherapeutic strategies as areas of active development in dogs, aligned with the broader movement toward personalized oncology.

Another set of contributions focuses on therapeutic strategies and treatment optimization, particularly in settings where durable local control or meaningful palliation remains difficult. Norquest et al. explored the combination of zoledronate and radiation therapy in canine osteosarcoma, integrating *in vitro* assessment with a prospective clinical cohort. Their work supports the feasibility and tolerability of the combined approach and raises important questions regarding optimal timing and biological variability in response. Also for canine osteosarcoma, Griffin et al. investigated clinical outcomes in dogs with axial and appendicular bone tumors treated with surgical stabilization combined with non-stereotactic radiation therapy. Their bi-institutional study demonstrates that this combined approach can achieve meaningful functional improvement, acceptable complication rates, and encouraging survival outcomes, even in advanced orthopedic oncology cases, supporting its role as a viable limb-sparing and palliative strategy. Petrucci et al. reviewed metronomic chemotherapy, integrating mechanistic concepts, including antiangiogenic and immunomodulatory effects, with available clinical data and practical considerations. Importantly, the authors clearly acknowledge the current limitations of the evidence, reinforcing the need for well-designed prospective studies while providing clinicians with a balanced overview of how metronomic approaches may be incorporated into practice.

Amaral et al. addressed the interface between nutrition and oncology in dogs and cats. By integrating evidence on cancer-associated metabolic changes, cachexia, the role of obesity, and nutritional interventions and supplementation, this review positions nutrition as a core element of supportive oncology care, contributing to functional preservation, quality of life, and improved treatment tolerance.

Therapeutic advancement is also dependent on establishing shared standards. In this context, Polton et al. provided a comprehensive consensus and guideline document for melanoma in dogs and cats, addressing staging, prognostic features, treatment modalities, and areas of uncertainty. By consolidating current knowledge and expert guidance, this contribution offers a pragmatic framework for clinicians managing one of the most biologically complex and clinically challenging tumor types in companion animals. Another key therapeutic challenge in veterinary oncology is the implementation of strategies that are biologically rational, clinically safe, and realistically applicable.

Taken together, these contributions illustrate how veterinary oncology is advancing through multiple, interconnected fronts, including refinement of local and systemic therapies, consensus building and clinical standardization, development of enabling immunology tools, and the steady expansion of minimally invasive diagnostics and biomarker research. At the same time, the Research Topic clearly reflects the field's ongoing challenges, such as biological heterogeneity, limited high-level evidence for some interventions, and the need for robust, clinically deployable diagnostic strategies. By presenting both progress and limitations in a balanced manner, this Research Topic aims to support clinicians and researchers working to deliver more precise, evidence-informed, and patient-centered veterinary cancer care.
